# Feasibility of Contrast-Enhanced Ultrasound Fusion With Pretreatment MR/CT for Recurrence Detection in Renal Cell Carcinoma Patients During Post-Ablation Surveillance

**DOI:** 10.1016/j.ultrasmedbio.2025.04.021

**Published:** 2025-05-28

**Authors:** Aylin Tahmasebi, Tania Siu Xiao, Corinne E. Wessner, Cristina M. Kuon Yeng Escalante, Andrej Lyshchik, Flemming Forsberg, Ji-Bin Liu, Christopher G. Roth, Shuchi K. Rodgers, Leann M. Kania, Kevin Anton, Anju Yadav, Costas D. Lallas, Stephen R. Topper, Edouard J. Trabulsi, John R. Eisenbrey

**Affiliations:** a Department of Radiology, Thomas Jefferson University, Philadelphia, PA, USA; b Department of Radiology, University of Pennsylvania, Philadelphia, PA, USA; c Department of Medicine, Johns Hopkins University, Baltimore, MD, USA; d Department of Urology, Thomas Jefferson University, Philadelphia, PA, USA; e Department of Urology, Jefferson Einstein Medical Center, Philadelphia, PA, USA

**Keywords:** Contrast-enhanced ultrasound—CEUS, Renal cell carcinoma, Cryoablation, Microwave ablation, Fusion imaging, Surveillance imaging, Ultrasound, Contrast agent

## Abstract

**Objective:**

Contrast enhanced ultrasound (CEUS) is a cost-effective, safe, and accurate modality for monitoring renal cell carcinoma (RCC) recurrence following percutaneous ablation. However, ultrasound delineation of treated tumor borders can be challenging post-ablation. Here, we demonstrate the feasibility of using post-treatment CEUS fused to preablation MR/CT to detect RCC recurrence during long term follow up.

**Methods:**

This study was performed as part of a larger ongoing prospective clinical trial for patients with biopsy-proven RCC treated with percutaneous ablation and receiving contrast enhanced (CE) -CT or -MRI for treatment response monitoring. CEUS with preablation CT/MR fusion was performed within 4 weeks of recurrence screening, and CE-CT/MRI was used as the reference standard. After intravenous injection of an ultrasound contrast agent, CEUS imaging of a single target lesion was performed with ultrasound fused to the patient’s pretreatment CT or MRI. RCC recurrence was diagnosed at the bedside based on the presence of iso- to hyper-enhancement within the margins of the ablation cavity compared to normal renal parenchyma.

**Results:**

To date, 50 participants have been recruited (76% male and 24% female). All participants tolerated the contrast injections without adverse events. CEUS was successfully fused to the participant’s pretreatment cross-sectional imaging in all cases and was found to aid in the delineation of the original treatment zone. Additionally, CEUS correlated perfectly (100% agreement) with CE-CT/MRI findings for all the participants.

**Conclusion:**

Preliminary results demonstrate that post-treatment CEUS fused with a pretreatment CT/MRI is feasible and may aid in the correct localization of the treated tumor margins during long-term post-ablation monitoring.

## Introduction

Renal cell carcinoma (RCC) is the most common type of kidney cancer in adults, accounting for 80%–85% of all primary renal neoplasms and is the 13^th^ most common cause of cancer death worldwide [1,2]. RCC is often found incidentally on imaging. A minimally invasive treatment option for T1a renal cancer (≤4.0 cm), which accounts for approximately 25% of all RCCs, is percutaneous thermal ablation [3,4]. Imaging surveillance following ablation typically includes abdominal contrast enhanced (CE) computed tomography (CT) or magnetic resonance imaging (MRI) after 3–6 months. Long-term surveillance utilizes annual CE-CT or -MRI (preferred) or ultrasound for 5 years or longer as clinically indicated [5]. However, a significant proportion of these patients have a contraindication to conventional contrast and serial CE-CT/MRI due to chronic kidney disease, nephrectomy status, contrast allergy or kidney transplants [6–8]. Although the new generation of Gadolinium agents has a better safety profile and requires smaller doses, they may still not be universally available or applicable for patients who need serial imaging during surveillance [9]. Additionally, these procedures often incur substantial cost and administrative barriers, such as preauthorizations, which occasionally present obstacles for these patients. Contrast enhanced ultrasound (CEUS), which uses stabilized gas microbubbles as a contrast agent is an ideal tool for post-ablation recurrence monitoring in this patient population. CEUS can alleviate challenges such as body habitus precluding MRI in some cases and degrading CT image quality through photon starving and higher kVp technique. CEUS like B-mode imaging has depth limitations due to attenuation [10]. Other challenges with CT and MRI include breath holding compliance leading to poor image quality, claustrophobia, and implanted devices, which may preclude MRI entirely or cause MRI or CT artifacts limiting lesion evaluation. Ultrasound contrast agents, unlike intravascular iodinated- and gadolinium-based agents, do not diffuse out of circulation, provide potential cost savings, and can be injected repeatedly to increase diagnostic confidence when necessary [11,12]. CEUS also provides better spatial and superior temporal resolution compared to MRI and CT, mitigating the occasional contrast-timing errors that occur with other modalities and providing a better visual illustration of contrast enhancement [13,14]. Specific imaging software for CEUS is commercially available across most manufacturers. This includes nonlinear imaging pulses to maximize ultrasound contrast agent signal generation and low mechanical index (MI; typically < 0.3) scanning to minimize microbubble destruction.

Fusion imaging allows for real-time and synchronous association of ultrasound images with one or more cross-sectional studies such as CT, MR, or positron emission tomography (PET), which are instantly reconstructed to fuse in the corresponding ultrasound plane. Fusion imaging can also be associated with other imaging technologies such as elastography, color Doppler and CEUS for better localization and characterization. While CEUS fusion to other imaging modalities is still experimental in renal studies, it has been widely used in the liver for both treatment guidance and post-ablation monitoring [15–20].

Our group has previously demonstrated the excellent sensitivity of CEUS for detecting RCC recurrence post-ablation [21,22]. However, the specificity was suboptimal given poor delineation of treated tumor boundaries and difficulties identifying the treatment cavity margins. Consequently, the goal of this work was to evaluate the feasibility of using post-treatment CEUS fused to a patient’s preablation CT/MR to detect RCC recurrence.

## Materials and methods

As part of this institutional review board approved ongoing trial, all participants provided written informed consent to undergo a series of CEUS exams within 4 weeks of a clinically scheduled CE-CT/MRI. Standard of care surveillance imaging CE-CT/MR exams were collected at approximately 6- and 12-month intervals as part of a larger ongoing clinical trial (NCT05641935) investigating the use of CEUS for RCC recurrence screening.

The study enrolled patients with biopsy-proven RCC, aged 18 years or older, who had undergone prior treatment with either microwave ablation (MWA) or cryoablation therapy. Patients were consecutively approached and excluded if they were pregnant, terminally ill, medically unstable, or had a known allergy to components of ultrasound contrast agents. All patients were confirmed to have available MR or CT imaging prior to the ablative therapy. CEUS scans were compared with reference standard cross-sectional imaging combined with biopsy confirmation in cases of diagnosed recurrence.

In this study, scanning was performed by three trained sonographers with extensive CEUS experience using a Logiq E10 scanner (GE HealthCare, Waukesha, WI, USA) with a C1–6 probe and Lumason (Bracco Diagnostics, Monroe Township, NJ) as the ultrasound contrast agent.

Fusion imaging was performed with a magnetic console placed approximately 5–12 cm above the patient’s subxiphoid region, while the patient was in either a supine or lateral decubitus position (depending on the lesion location). The magnetic field was then used to recognize the ultrasound transducer and create 3D spatial position and orientation [23]. Digital Imaging and Communications in Medicine (DICOM) datasets of the patient’s pretreatment noncontrast CT or MRI (3 months to 7 years prior to CEUS) were retrieved directly from the Picture Archiving and Communication System (PACS) and uploaded manually onto the ultrasound scanner. Once the images were uploaded, the real-time ultrasound images and prior cross-sectional imaging could be viewed side-by-side or in an overlay mode [24]. The sonographer selected three points in the kidney that were of interest and matched each of these points to the corresponding noncontrast CT or MR images to fuse the imaging plane. Our study team found that incorporating the upper and lower margins of the kidney in a longitudinal plane instead of arbitrary upper, mid, and lower pole regions resulted in a more accurate fusion. An additional co-registration point was then added if an anatomical landmark was present in both imaging modalities. Once the ultrasound and CT/MR examinations were fused and optimized, CEUS was ready to be performed using the coded harmonic imaging contrast package on the scanner.

An intravenous catheter was placed in the patient’s upper extremity by a registered nurse for the Lumason injection. A low MI of <0.13 was used to minimize microbubble destruction during imaging. Similarly, no flash modes or destructive pulses are employed during CEUS exams. The gain settings were adjusted to minimize nonlinear signals prior to contrast injection. The focal zone was placed at or immediately below the approximate depth of the ablation cavity to maximize the generation of nonlinear signals during CEUS. With the cross-sectional imaging acquisition already fused with the ultrasound (VolumeNav, GE HealthCare), patients received a bolus of 1.0–1.6 mL of Lumason followed by 10 mL of normal saline. Real-time CEUS was performed with fused cross-sectional MR/CT. During the early phase of CEUS, the tumor midline was imaged until complete renal enhancement was observed (approximately 30 seconds post-injection), followed by imaging sweeps to evaluate the entire treatment cavity. Sweeps were acquired and imaging continued until either the onset of contrast washout in the renal parenchyma was observed or 90 seconds after the contrast injection if no definite contrast washout was observed. The ablated cavity was intermittently imaged until near complete contrast dissipation was determined 5–7 minutes after contrast injection.

To evaluate the feasibility and demonstrate proof of concept, bedside interpretation of the CEUS findings was made by the study team based on the presence of arterial phase iso- or hyper-enhancement within the ablation cavity being indicative of a recurrent tumor. These findings were compared to the cross-sectional imaging findings obtained as the standard of care.

## Results

To date, 50 cases (76% male and 24% female) have been successfully enrolled and scanned. The average participant age was 70.7 ± 9.8 years. Ablative treatment of the RCC consisted of MWA (52%) or cryoablation (48%). The average time between post-treatment CE-CT/MRI (used as the reference standard) and CEUS was 9.4 ± 8.4 days. The average time between ablation and CEUS was 30.5 ± 23.1 months (range 1.2–87 months).

All participants tolerated the contrast injections with no adverse events. Contrast enhancement of the surrounding renal parenchyma was observed in all patients approximately 20–25 seconds post-injection and lasted roughly 5 minutes before full washout.

Of the examined patients, only 1 patient (1/50 patients, 2%) to date has demonstrated hyper-enhancement within the treatment cavity, indicating RCC recurrence. The recurrence was also detected on an MRI and later confirmed by pathology. The other 49 cases (49/50 patients, 98%) showed no evidence of recurrent or residual disease by CEUS which matched with the CE-CT/MRI findings. These preliminary CEUS results suggest 100% agreement among the 50 recruited patients to date in identifying RCC recurrence during post-ablation surveillance.

**Figure 1.**
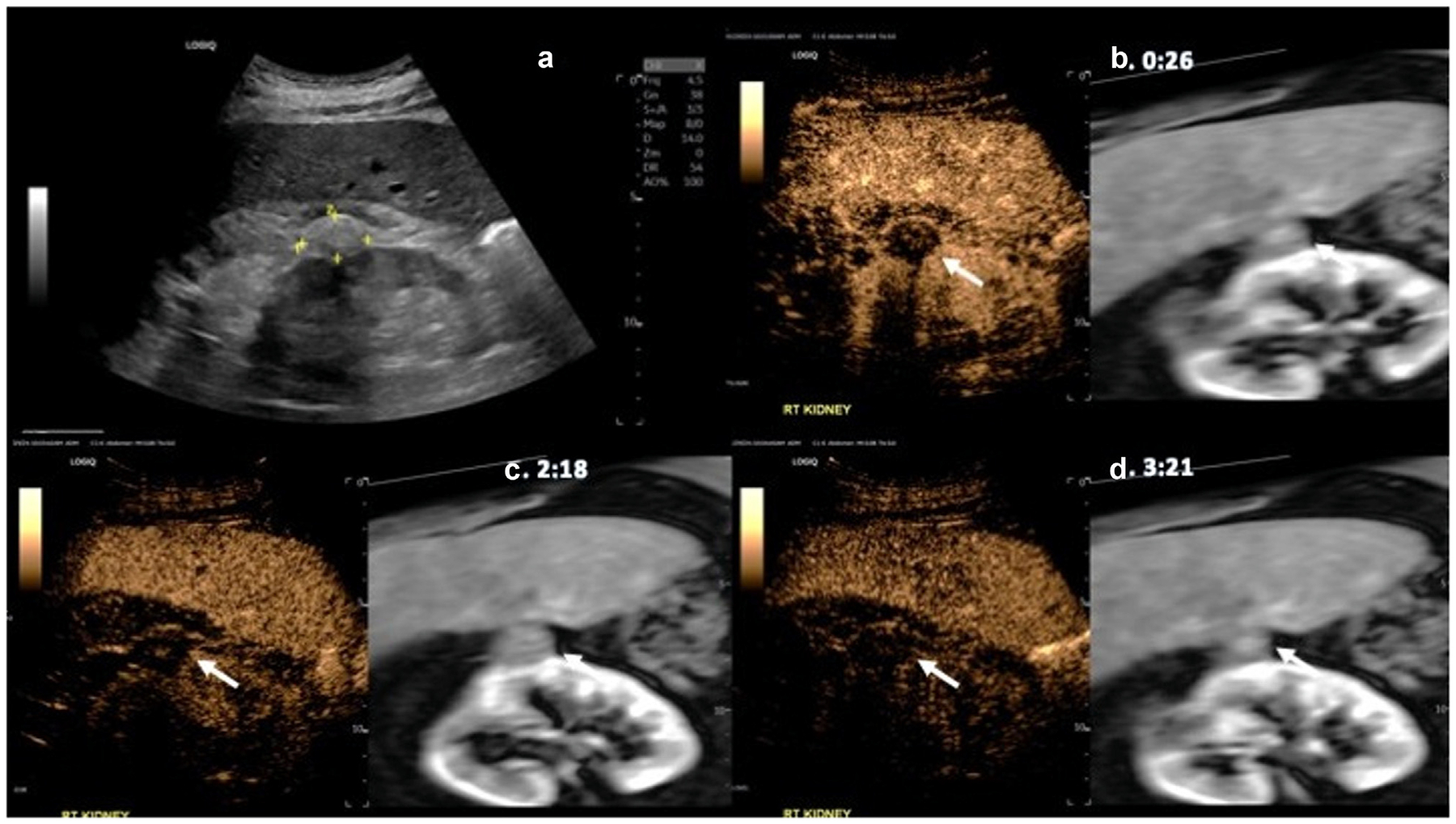
B-mode and fusion imaging of CEUS and a patient’s preablation MRI. (a) Displays an example B-mode image of the previously ablated cavity. (b) An example image from the arterial phase at 26 seconds post-injection showing no intratumoral enhancement. (c) Example image from the beginning of the renal washout at 2:18, and (d) Near complete renal washout at 3:21.

**Figure 2.**
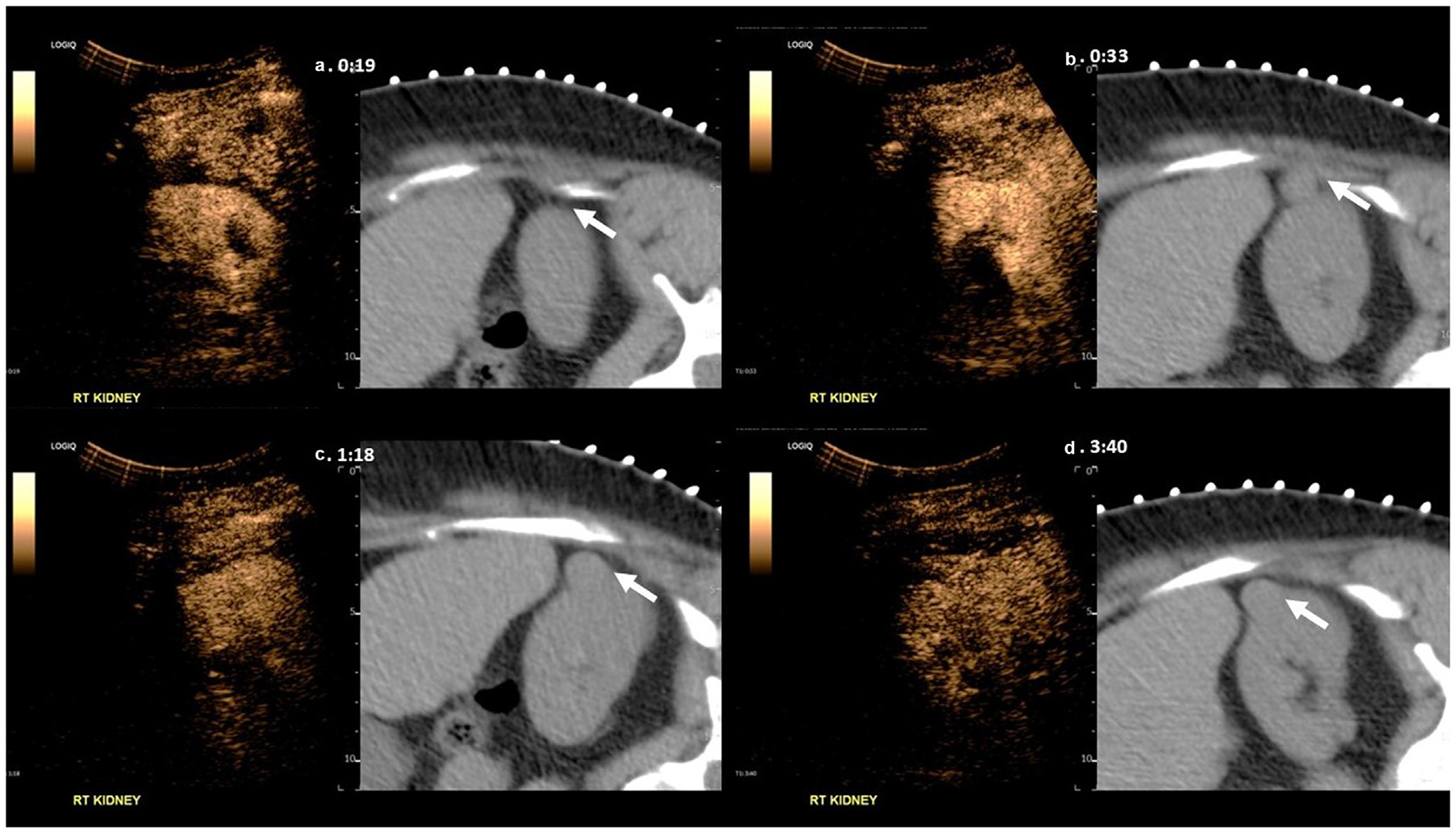
An example of a challenging case in which the region of interest on CEUS did not correctly match the patient’s preablation CT for fusion imaging. Note, that while the tumor is visualized well on the pretreatment MRI, the ablation cavity is not well appreciated on CEUS.

**Figure 3.**
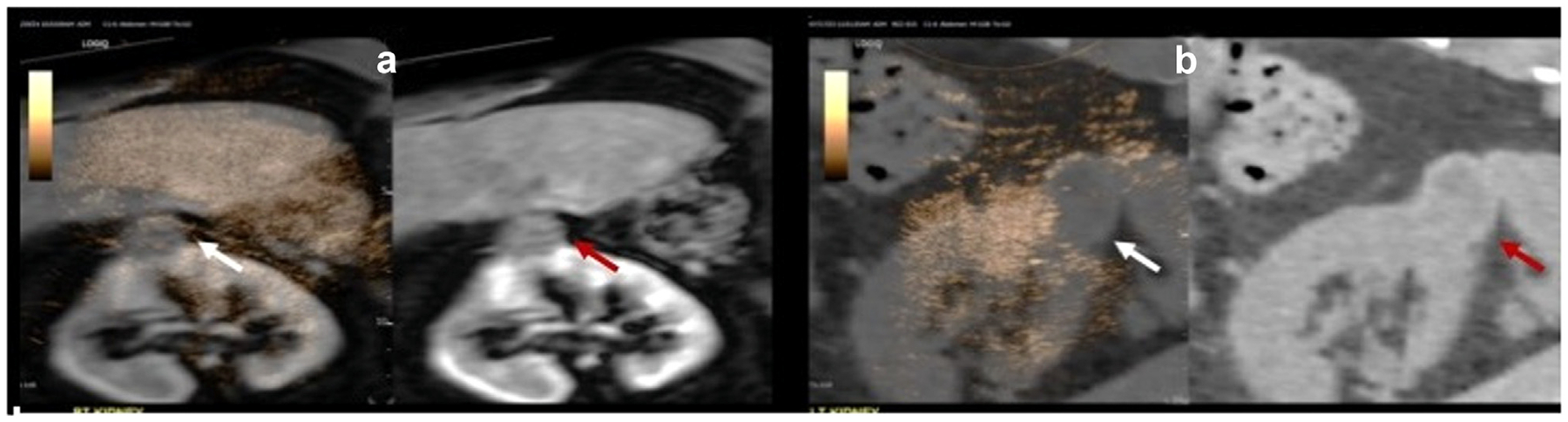
(a) An example of an excellent overlay of patient’s CEUS and preablation MRI from 4 years prior. (b) An example of a challenging fusion of patient’s CEUS and preablation CT three years prior.

Fusion of real-time CEUS with the patient’s pretreatment CT/MRI was found to be feasible, albeit with varying degrees of registration alignment. Figure 1a displays a B-mode image of the ablation cavity and accurate fusion alignment of CEUS and MRI (Fig. 1b, 1c, 1d). Tracking through the region of interest following fusion demonstrated consistent and accurate fusion of both the treatment cavity and surrounding kidney. Alternatively, Figure 2 demonstrates a challenging case in which the region of interest on CEUS and CT did not correctly match. This was a failed case in which the treated tumor cavity could not be well visualized on CEUS because of artifacts from rib shadowing and scanning challenges due to the patient’s larger body habitus. Figure 3 displays examples of accurate and poor fusion alignment of CEUS and preablation MRI in two participants. In both cases, pretreatment cross-sectional imaging was obtained 3 years prior to CEUS.

**Figure 4.**
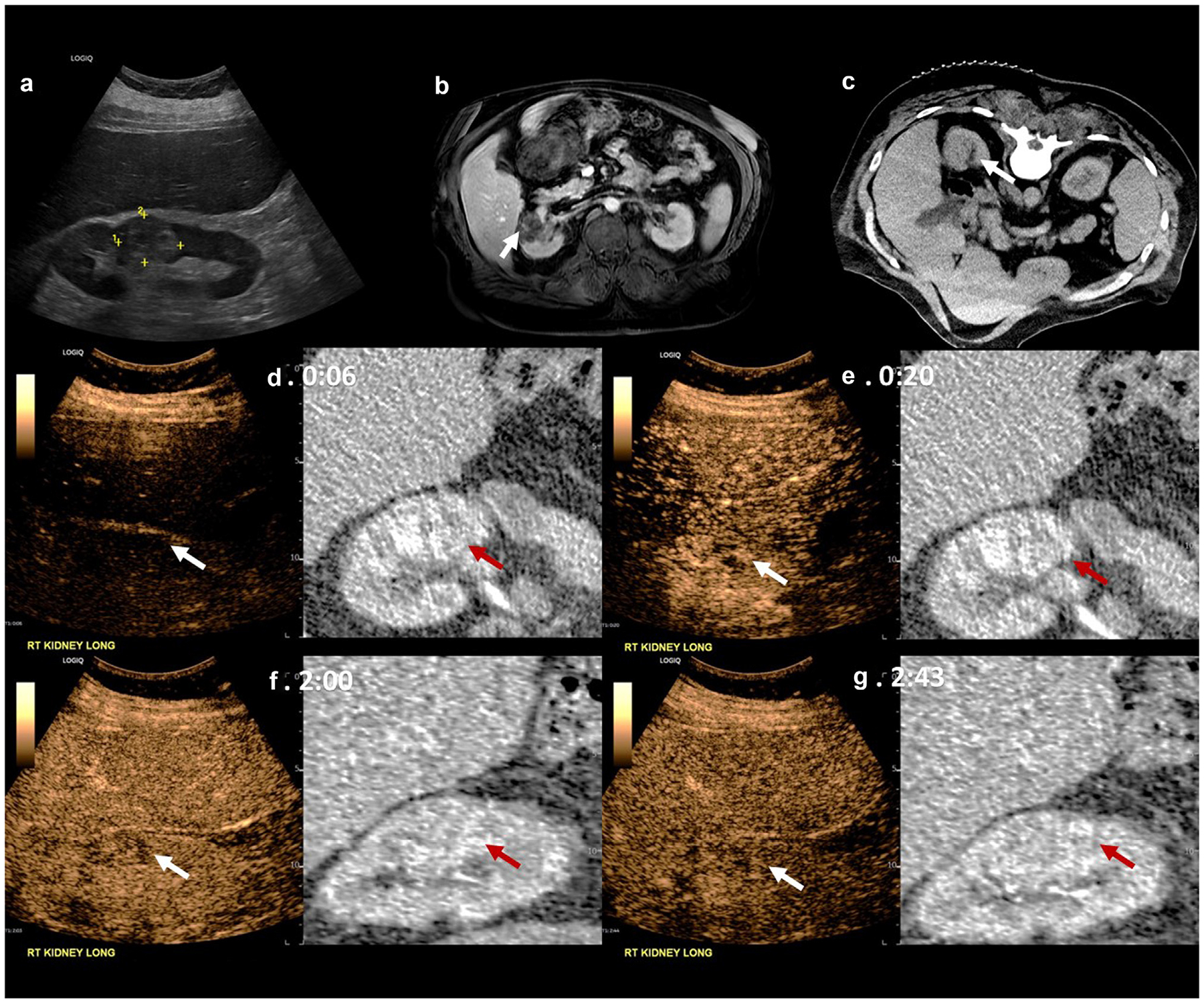
B-mode, MRI, CT and CEUS fusion imaging of a patient with disease recurrence. (a) Displays patient’s ultrasound B-mode image of the recurrent lesion in the previously ablated location with dimensions of 3.8 cm × 2.9 cm, (b) Patient’s most recent CE-MRI displaying the recurrent lesion (c) Displays patient’s preablation CT in, which was used for fusion imaging three years later Example of CEUS with fusion acquired (d) 6 seconds post-injection, (e) 20 seconds post-injection showing the arterial phase iso-enhancement, (f) 2 minutes post-injection at the start of wash out, and (g) 2 minutes and 43 seconds post-injection showing early mild washout.

Figure 4 presents imaging of a case of post-ablation recurrence, in a 63-year-old male participant with a history of clear cell RCC and cryoablation 3 years prior to CEUS imaging. Recurrence was detected on both CE-MRI (Fig. 4b) and CEUS (Fig. 4d, 4e, 4f, 4g) and confirmed as a recurrence by tissue sampling obtained during retreatment. While based on a very limited sample size, this study demonstrates the potential of CEUS to diagnose cases of RCC recurrence.

**Figure 5.**
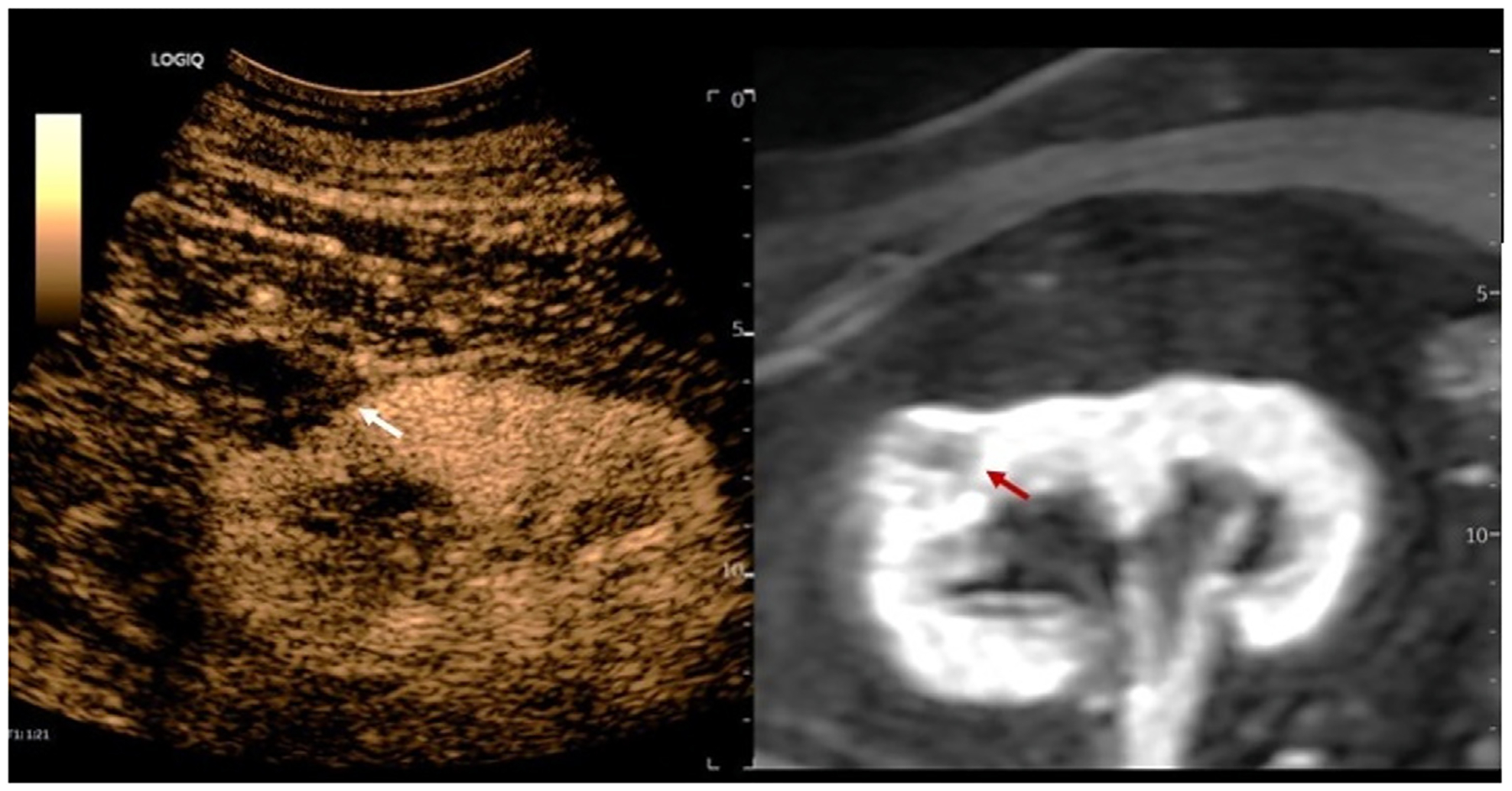
Example of a well-fused case but with the potentially misleading appearance of intratumoral enhancemnt. In the case of residual or recurrent tumor, contrast enhancement is seen in the arterial phase. In this case, intratumoral enhancement was static, which indicates an artifact likely from tissue necrosis.

In some cases, pseudo-enhancement or delayed hypo-enhancement was observed, but this was not indicative of true RCC recurrence. Examples of this delayed enhancement are provided in Figure 5, reflecting the importance of using dynamic imaging to rule out static artifacts.

## Discussion

This study demonstrates the feasibility of fusing post-ablation CEUS to preablation MRI/CT to improve the delineation of the tumor boundary based on 50 cases. This ongoing clinical trial plans to recruit 210 cases and if successful, may reduce the number of cross-sectional imaging exams required for RCC recurrence surveillance following percutaneous ablation. This approach is possible given the relative stability of both the normal renal parenchyma and the ablation cavity for years following treatment [25].

CEUS with and without fusion has been used in renal tumor studies mainly for renal mass classification [16,24,26–31], intervention guidance [32–35], and treatment response evaluation [36–47]. Our 100% concordance between CE-CT/MR and CEUS supports previous studies, which also reported high inter-modality concordance with CEUS after percutaneous RFA or cryoablation. Kong et al. performed paired CEUS and CE-CT 1 month after RFA in 64 RCCs to evaluate ablation success. Among 62 complete and 2 incomplete ablations, concordance between imaging modalities was 100% [44]. Similarly, Meloni et al. compared CEUS to CE-CT/MRI retrospectively in 28 RCCs after RFA and observed concordance among 27 of 28 tumors. CEUS failed to detect tumor recurrence in 1 tumor, which was noted preprocedurally to be hypovascular and minimally enhancing on CE-CT [38]. Allard et al. also found perfect concordance between CEUS and CE-CT (kappa = 1.0; *p* < 0.0001) with a diagnostic rate of 94.6% in post-RFA surveillance of renal tumors [39]. Their group suggested CEUS for post-ablation surveillance, should always have a pretreatment CE-CT/MR of the lesion, which is identical to our study design fusing the preablation cross-sectional imaging with CEUS.

Eisenbrey et al. studied harmonic and subharmonic CEUS for renal mass characterization and post-cryoablation treatment response in 12 tumors. Harmonic and subharmonic CEUS showed accuracies of 75% and 83%, respectively in predicting treatment outcome [21]. Zeccolini et al. also reported 100% concordance between CEUS and CE-MRI in surveillance of 25 small renal tumors treated by cryoablation [42]. In a study by Li et al., CEUS showed accuracy of 97.6% in long-term surveillance post-MWA in comparison to CT/MRI [46]. Calio et al. studied the efficacy of 2D and 3D CEUS for recurrence detection in RCC post-ablation surveillance. CEUS successfully identified all cases of recurrence by enhancement of the full ablation cavity with equal arrival times and intensities compared to the renal cortex. They mentioned that some patients with complete response to treatment developed delayed enhancement at the periphery of the ablation cavity over time, which corresponds to fat necrosis, scarring or granulation tissue within the ablation cavity. In this study, early enhancement was a criterion for recurrence of disease [22]. Chandrasekar et al. proposed fractional velocity, a metric extracted from 2D and 3D CEUS data for risk stratification and malignancy prediction of complex renal cysts [48]. Eisenbrey et al. also retrospectively investigated the long-term impact in patients with indeterminate renal masses. CEUS provided definitive diagnosis in 71.6% of cases of which 15.6% underwent surgical resection with pathology proven malignancy in 87.5% of specimens [11]. Furthermore, King et al. studied different CEUS features in subtypes of RCC and found that peak enhancement greater than that of the renal cortex and rapid time to peak intensity were indicative of clear cell RCC, while hypo-enhancement with respect to the renal cortex and slower time to peak enhancement were more characteristic of papillary RCC [49]. The pseudo-enhancement observed in few cases in our study was also reported in another CEUS study assessing the therapeutic response of RCC to radiofrequency ablation (RFA) [44]. Yet another study evaluated the frequency of false positive enhancement after RCC ablation on CE-MR and found it occurs commonly during the first month post-ablation [50]. This indicates the importance of dynamic evaluations when screening for recurrence and waiting at least 3 months before initial imaging to evaluate treatment response.

While CEUS for RCC recurrence has shown promise in these preliminary studies, limitations and challenges should be addressed. Fusion can be challenging if the ideal ultrasound scanning plane is different from the classical orthogonal CT or MR image. When there is more than one imaging modality from different time points, co-registration must be performed to fuse the imaging modalities to create a matching 3D plane. Co-registration points are placed on all corresponding images at the exact locations to fuse the imaging modalities correctly. Since the ideal ultrasound scanning plane can be difficult to achieve in all patients, our group has found that adding multiple anatomical landmarks can improve fusion. For renal fusion imaging, studies have suggested choosing anatomical landmarks such as a renal artery or vein, hilum, a simple renal cyst or even the main portal vein in the liver for fusing multiple imaging modalities. However, our group has found that the most accurate anatomical landmark for renal fusion imaging is matching the upper and lower renal poles as well as an additional anatomical landmark (such as a renal cyst or hilum). In this clinical trial, preablation cross-sectional imaging was used. This delay in imaging results in longer intervals between the preablation CT/MRI and follow-up CT/MRI imaging (time of the CEUS examination), which may make co-registration using anatomical landmarks more challenging (for example, 7 years passed between post-treatment CEUS and preablation imaging for 2 of the cases in this study). Moreover, breathing and displacement of the abdominal structures due to previous abdominal surgeries or pressure from the ultrasound probe can affect the process of registration. CEUS, like conventional ultrasound imaging, is limited in obese patients as demonstrated in one case in this study (Fig. 2) [51]. Moreover, the efficacy of CEUS in deep sitting and hypovascular tumors needs future studies [38,39]. A crucial aspect of interpreting CEUS imaging is the recognition of artifacts and artifactual enhancement. Techniques such as destruction-replenishment may help differentiate true lesion enhancement from calcifications or fat in the ablated cavity. Adjusting the patient’s position and optimizing the acoustic window can also reduce the transducer-to-target distance, helping to eliminate intervening enhancing structures and mitigate these artifacts. Moreover, true enhancement will display dynamically moving microbubbles, while pseudo-enhancement will present stationary microbubbles [52].

Lastly, this study was designed to calculate inter-modality agreement between CEUS and CE-CT/MR, but not other test characteristics such as sensitivity, specificity, and positive or negative predictive values. We cannot definitively rule out false negative results among CEUS and CE-CT/MR, since renal biopsy was not performed in patients with negative CE-CT/MR. Also, histopathology confirmed RCC recurrence in one patient with arterial enhancement, excluding the possibility of false positive results from either modality.

CEUS is a cost-effective and safe imaging modality for detecting recurrent or residual RCC following percutaneous ablation. The role of renal CEUS has previously demonstrated important cost-savings and direct benefit to patient management in several retrospective studies [11,12]. Additionally, CEUS provides improved temporal and spatial resolution, and no concerns of nephrotoxicity. However, it is not suitable for whole-body imaging and may not be ideal for surveillance of new tumors, suggesting an optimal surveillance strategy could include alternating modalities. Our future studies from this ongoing clinical trial will include a larger sample size with an extended follow-up period and readings of both modalities to fully evaluate these approaches.

## Conclusion

Initial results demonstrate the feasibility of using post-treatment CEUS fused with pretreatment CT/MRI for monitoring RCC recurrence post-ablation. Preliminary results show complete concordance between CEUS and CE-CT/MRI for detecting RCC recurrence following percutaneous ablation and fusion with pretreatment CT/MRI was found to be useful for correct localization of the prior tumor boundaries on CEUS.

## Data Availability

All data are available upon request.
